# Review and Analysis of Existing Mobile Phone Apps to Support Heart Failure Symptom Monitoring and Self-Care Management Using the Mobile Application Rating Scale (MARS)

**DOI:** 10.2196/mhealth.5882

**Published:** 2016-06-14

**Authors:** Ruth M Masterson Creber, Mathew S Maurer, Meghan Reading, Grenny Hiraldo, Kathleen T Hickey, Sarah Iribarren

**Affiliations:** ^1^ Columbia University School of Nursing New York, NY United States; ^2^ College of Physicians & Surgeons Columbia University New York, NY United States; ^3^ NewYork-Presbyterian/Columbia University Medical Center Division of Cardiology New York, NY United States

**Keywords:** mobile apps, mobile health, heart failure, self-care, self-management, review, symptom assessment, nursing informatics

## Abstract

**Background:**

Heart failure is the most common cause of hospital readmissions among Medicare beneficiaries and these hospitalizations are often driven by exacerbations in common heart failure symptoms. Patient collaboration with health care providers and decision making is a core component of increasing symptom monitoring and decreasing hospital use. Mobile phone apps offer a potentially cost-effective solution for symptom monitoring and self-care management at the point of need.

**Objective:**

The purpose of this review of commercially available apps was to identify and assess the functionalities of patient-facing mobile health apps targeted toward supporting heart failure symptom monitoring and self-care management.

**Methods:**

We searched 3 Web-based mobile app stores using multiple terms and combinations (eg, “heart failure,” “cardiology,” “heart failure and self-management”). Apps meeting inclusion criteria were evaluated using the Mobile Application Rating Scale (MARS), IMS Institute for Healthcare Informatics functionality scores, and Heart Failure Society of America (HFSA) guidelines for nonpharmacologic management. Apps were downloaded and assessed independently by 2-4 reviewers, interclass correlations between reviewers were calculated, and consensus was met by discussion.

**Results:**

Of 3636 potentially relevant apps searched, 34 met inclusion criteria. Most apps were excluded because they were unrelated to heart failure, not in English or Spanish, or were games. Interrater reliability between reviewers was high. AskMD app had the highest average MARS total (4.9/5). More than half of the apps (23/34, 68%) had acceptable MARS scores (>3.0). Heart Failure Health Storylines (4.6) and AskMD (4.5) had the highest scores for behavior change. Factoring MARS, functionality, and HFSA guideline scores, the highest performing apps included Heart Failure Health Storylines, Symple, ContinuousCare Health App, WebMD, and AskMD. Peer-reviewed publications were identified for only 3 of the 34 apps.

**Conclusions:**

This review suggests that few apps meet prespecified criteria for quality, content, or functionality, highlighting the need for further refinement and mapping to evidence-based guidelines and room for overall quality improvement in heart failure symptom monitoring and self-care related apps.

## Introduction

Heart failure (HF) is a common, complex, and costly cardiovascular condition. Heart failure currently affects 5.7 million Americans [1], is the fastest growing cardiovascular condition in the United States [2], and the most common reason for hospitalization among older adults [3-6]. Worldwide, the prevalence of HF is estimated to be more than 23 million people [7]. The most common reason for HF-related hospitalizations is symptom exacerbations. Symptom changes are often insidious, making it difficult for patients to recognize and respond to changes early and resulting in need for hospital-based management of HF exacerbations. To reduce the societal and cost burden of HF, effective symptom management strategies are important for patients and also may help to reduce hospital admissions [8,9]. Major clinical guidelines recommend the inclusion of daily symptom monitoring as part of routine management of patients with HF [10].

With an uptake of mobile phone ownership among adults in the United States [11], there is growing opportunity to capitalize on the use of mobile phone technology to enhance the management of HF. Mobile phones are an optimal vehicle for housing mobile health (mHealth) apps for symptom monitoring because they are accessible continuously, portable, and convenient. Mobile health apps are reported to be an ideal platform for behavior change because of popularity, connectivity, and increased sophistication [12]. Apps can support added functionalities and have the potential for real-time data collection, graphic feedback, interactivity, and links to social functionalities [12]. In addition, apps have the potential to be useful for symptom management because they can include behavioral prompts, reminders, illness monitoring, and self-management programs that extend far beyond the clinic walls.

Currently, reviews of commercial mHealth apps exist to support patients undergoing bariatric surgery [13], those who are managing bipolar disorder [14], cancer [15], cardiovascular disorders [16], chronic pain [17,18], depression [19], diabetes [20], health care–associated infection prevention [21], human immunodeficiency virus [22,23], and schizophrenia [24]. A review has been conducted on published literature on mHealth apps for HF [25]; however, it did not include an evaluation of commercially available mHealth apps. To date no studies have assessed commercially available apps to support HF symptom monitoring and self-care. To address this gap, we conducted a thorough review of commercially available existing mobile apps focused specifically on self-management and symptom monitoring for patients with HF. Our objectives were to (1) identify HF-related apps available in the main app stores; (2) describe their characteristics; (3) identify if any of the available apps have been rigorously tested; and (4) rate the quality of the apps based on the Mobile Application Rating Scale (MARS) [26], functionality score from the IMS Institute for Healthcare Informatics report [27], and Heart Failure Society of America (HFSA) guidelines for nonpharmacologic management [10].

## Methods

### Systematic Search Criteria and Selection

In January 2016, we conducted a thorough review of mobile apps across 3 mobile app stores: Apple iTunes Store, Android Google Play store, and Amazon Appstore. The following search terms were included: “heart failure,” “cardiology,” “heart failure and self-management,” “heart failure and symptom management,” “heart failure and symptom monitoring,” “heart failure and self-care,” “cardiology and symptom management,” “cardiology and symptom monitoring,” “heart,” “symptom,” “symptom management,” “self-care,” and “self-care and heart.” Each term was searched in each of the 3 app stores listed.

**Figure 1:**
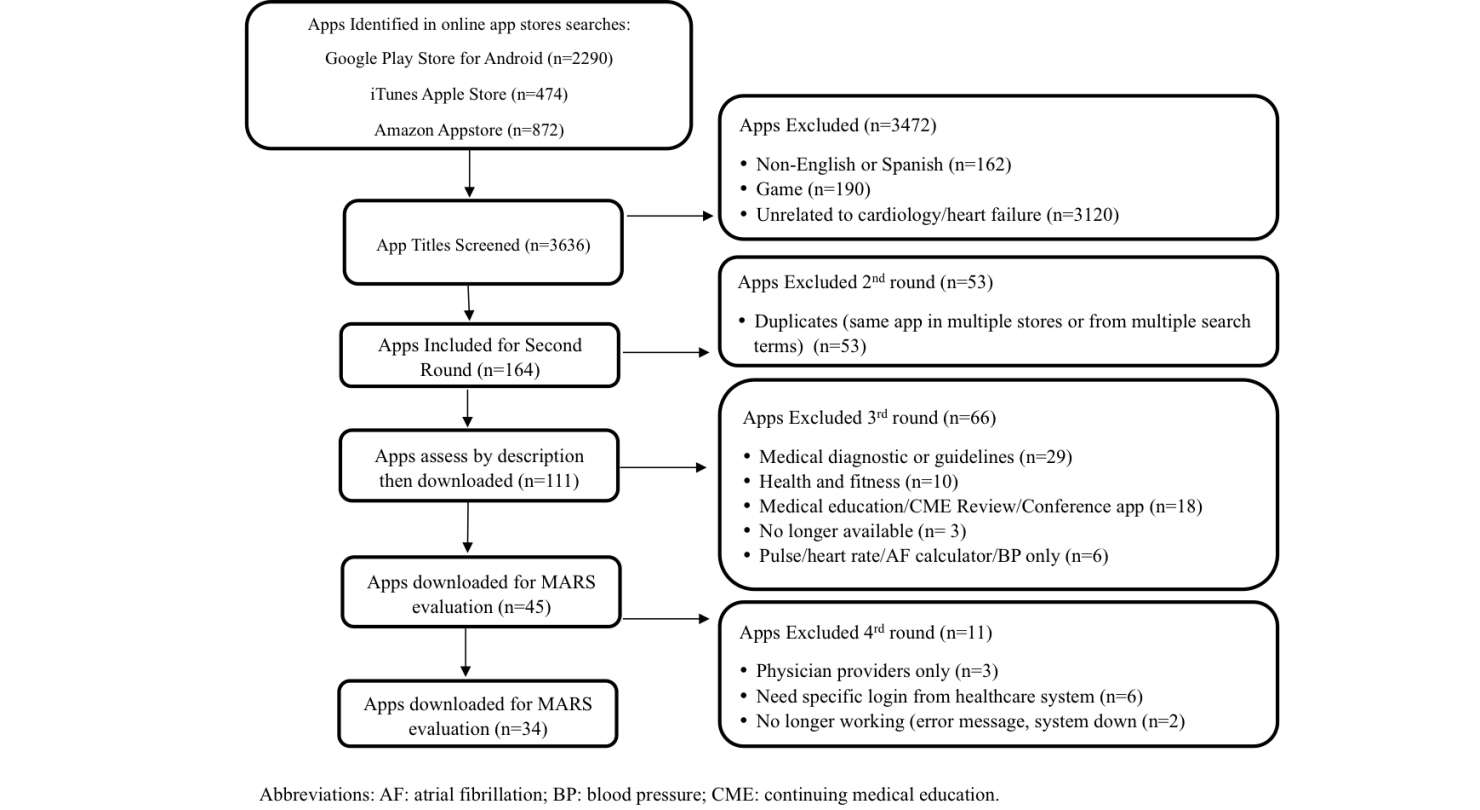
Screening process flowchart.

Preliminary screening was conducted based on app titles, full marketing description, and screenshots of the potential apps for relevance and inclusion. Apps were excluded if they were games, unrelated to health, or not written in English or Spanish. The second round of exclusion criteria focused on removing (1) duplicate apps (those found in multiple stores or from multiple search terms), (2) highly similar versions of the same app (eg, “lite” or “pro” versions), (3) apps that are not patient-facing, (4) apps focused solely on health and fitness, (5) apps for continuing medical education or conference apps, and (6) apps that were no longer available (Figure 1). Team members reviewed the apps after each round of exclusion criteria were completed (almost 70% of the apps were rated by at least two reviewers). The remaining apps were downloaded, reviewed (iOS 9.2.1 on iPhone 6; iPad mini or Android phone), rated, and evaluated by 2 reviewers (GH and RMC).

A data extraction form was built using a Google Docs survey that included the full MARS scale, IMS Institute for Healthcare Informatics functionality scoring system, and 8 questions related to specific self-care behaviors recommended in the “Nonpharmacologic Management and Health Care Maintenance in Patients with Chronic Heart Failure” published by the HFSA [10].

### Measures or Rating Tool

We rated and ranked the apps based on 3 scores: (1) MARS quality score [26], (2) IMS Institute for Healthcare Informatics functionality score [27], and (3) consistency with HFSA guideline recommendations [10] with an additional question related to the number of self-care behaviors that the apps addressed. The MARS was used to rate app quality and includes 3 sections and a modifiable app-specific section: classification, quality, and satisfaction [26]. The classification section provides descriptive information about the apps. The objective app quality section includes 19 items divided into 4 scales: engagement, functionality, aesthetics, and information quality. The subjective quality section contains 4 items evaluating the user’s overall satisfaction. MARS items are scored using a 5-point Likert scale (1-inadequate, 2-poor, 3-acceptable, 4-good, and 5-excellent). The final MARS scores include 4 subscale scores, a total mean score, subjective quality score, and an app-specific subscale that assesses perceived effect on the user’s knowledge, attitudes, and intentions to change as well as likelihood of changing the identified targeted behaviors.

The IMS functionality score is based on 7 functionality criteria and 4 functional subcategories as described in detail in the IMS Institute for Healthcare Informatics report [27] (Table 1). Each app was assessed for having or not having 11 functionalities and given a functionality score (0-11) [27].

**Table 1:** IMS Institute for Healthcare Informatics functionality scoring criteria.

Functionality scoring criteria		Description
**1. Inform**		Provides information in a variety of formats (text, photo, video)
**2. Instruct**		Provides instructions to the user
**3. Record**		Capture user entered data
	Collect data	Able to enter and store health data on individual phone
	Share data	Able to transmit health data
	Evaluate data	Able to evaluate the entered health data by patient and provider, provider and administrator, or patient and caregiver
	Intervene	Able to send alerts based on the data collected or propose behavioral intervention or changes
**4. Display**		Graphically display user entered data/output user entered data
**5. Guide**		Provide guidance based on user entered information, and may further offer a diagnosis, or recommend a consultation with a physician/a course of treatment
**6. Remind or Alert**		Provide reminders to the user
**7. Communicate**		Provide communication with HCP^a^/patients and/or provide links to social networks

^a^HCP: health care provider.

Two functionality scores were used for this review because the functionality scores provide different types of information on app functionality. The MARS functionality score focuses on performance, ease of use, navigation, and gestural design of the app [26], whereas the IMS Institute for Healthcare Informatics functionality score focuses on scope of functions, including informing, instructing, recording, displaying, guiding, reminding, and communicating information [27].

Each of the apps was also evaluated for whether it included 8 specific self-care behaviors recommended by HFSA guidelines [10]. These behaviors included daily weighing, checking extremities for swelling, doing physical activity or exercise, eating a low-salt diet, taking daily medications, attending doctor’s appointments, daily monitoring of HF symptoms, and actively responding to symptoms when they change, consistent with HFSA nonpharmacologic guidelines [10].

### Data Analysis

Four reviewers (GH, RMC, MR, and SI) watched the accompanying MARS instructional videos for how to use the MARS scale. Each reviewer rated 4 randomly selected apps to evaluate interrater reliability. The interclass correlation coefficients (ICCs) were calculated between the 4 reviewers. On the basis of ICC guidelines by Shrout and Fleiss [28], we selected an individual consistency-of-agreement intraclass correlation (CA-ICC) for a two-way random-effects model. The assumptions of this model include that the variance of raters only adds noise to the mean estimate and that the mean rater error is zero. It also models both the effect of the individual rater as well as the average of the raters and assumes both are drawn randomly from a larger population [29].

## Results

### Descriptive Characteristics

Android Google Play, Apple iTunes, and Amazon Appstore searches identified 3636 potentially relevant apps, of which 34 met our final inclusion criteria. The flow diagram (Figure 1) provides an overview of the selection process and categories for exclusion. Most apps were excluded because they were unrelated to HF (n=3120), not available in English or Spanish (n=162), or were games (n=190).

Table 2 provides the full list of the included apps and their characteristics. Most apps were free to download (31/34, 91%) with costs up to US $4.99. Most of the apps have been updated within the last year (63%). The average consumer star rating across all of the apps was 3 out of 5 with a range of 0 to 5. The number of individual ratings ranged from 0 to more than 52,000. Most of the apps had been installed between 100 and 500 times, but WebMD and iTriage had been installed between 5 and 10 million times. Almost 50% of the apps included a privacy policy.

**Table 2:** Description of included apps.

Name	Star rating	Installs^a^	Version	Cost, US $	Platform	Privacy policy	IMS score^b^
ASCVD^c^ Risk Estimator	3.5	N/R^d^	1.1	Free	Apple	No	6
AskMD	5	N/R	2.4.1	Free	Apple	No	9
BloodPressureDB	4.3	100-500K	5.64.0	Free	Google	Yes	7
Cardiograph	3.8	100-500K	3.2	Free	Google	Yes	5
Continuous Care Health App	3.9	100-500K	2.2.6	Free	Apple & Google	Yes	11
FAQs in Heart Failure	4.3	1-5K	1.2	Free	Google	No	2
Health Manager	3.5	N/R	2.1	$4.99	Apple	Yes	7
Healthy Ally	0	10-50	1.3.4	Free	Google	Yes	6
Healthy Heart	0	10-50	1.0.2	Free	Google	No	1
Healthy Heart Numbers	0	N/R	1	$2.99	Amazon	No	2
Heart Disease	3.3	500-1000	2.3.3	Free	Google & Amazon	No	1
Heart Disease & Symptoms	3.7	500-1000	1	Free	Google	No	0
Heart Failure Health Storylines	0	100-500	2.2.6	Free	Apple & Google	Yes	10
Heart Guide	0	100-500	1	Free	Google	Yes	4
Heartkeeper	4.7	1-5K	1.3	Free	Google	Yes	7
Heart Log	2	N/R	1.2	Free	Apple	No	5
Heart Services	3.8	100-500K	1.7.3	Free	Google	No	3
iTreat-Medical Dictionary	4.4	5-10K	1	Free	Apple & Google	Yes	4
iTriage	4.5	5-10 million	5.26	Free	Apple & Google	Yes	9
mediSOS	5	100-500	1.12	Free	Google	Yes	4
Miniatlas Hypertension	0	N/R	4	$1.99	Apple	No	3
My Cardiologist	5	100-500	1	Free	Google	No	2
My Health Tracker	2.7	N/R	1	Free	Amazon	No	3
My Heart Rate Monitor & Pulse Rate	4	N/R	1.3	Free	Apple	No	5
MyHeartApp	5	N/R	1.5	Free	Apple	No	4
Pulse Pro	3	N/R	1.2.4	Free	Apple	Yes	4
REKA	4.6	500-1000	2.0.5	Free	Google	Yes	3
SelfCare-My Health Record (MHR)	0	N/A^d^	1	Free	Apple	No	5
Symple	4.5	N/R	2.0.6	Free	Apple	Yes	11
Track your Heart Failure Zone	0	N/R	1	$1.99	Amazon	No	2
Urgent Care 24/7	4	N/R	1.1	Free	Apple	Yes	9
URI Life	4.6	1-5K	1	Free	Google	Yes	6
WebMD	4.5	5-10 million	5.9.3	Free	Apple & Google	No	11
WOW ME 2000mg	4	100-500	1.1	Free	Apple & Google	No	7

^a^Data on number of installs were only available in Google Play.

^b^The IMS score is the IMS Institute for Healthcare Informatics functionality score ranging from 0-11.

^c^ASCVD: atherosclerotic cardiovascular disease

^d^N/R: not recorded.

^d^N/A: not applicable.

### MARS App Quality Scores

Table 3 presents the 4 subscale scores (engagement, functionality, aesthetics, and information), overall quality score, subjective quality score (satisfaction), and app-specific health behavior score from the MARS. It was not possible to rate item 19, because a PubMed search identified only 3 efficacy studies among the 34 apps. More than 2/3 of the apps were evaluated by 2 or more expert MARS raters, and there was excellent interrater reliability (two-way mixed CA-ICC=.93, 95% CI: 0.68-0.99). Of the 4 subscales, functionality had the highest score and median engagement had the lowest (2.9).

The median overall MARS score was 3.4 out of 5, and 68% (23/34) had a minimum acceptability score of 3.0. Overall, the AskMD app had the highest average MARS total (4.9) followed by WebMD (4.4), Symple (4.3), Heart Failure Health Storylines (4.1), and ContinuousCare Health App (4.0). Heart Failure Health Storylines (4.6) and AskMD (4.5) had the highest scores for behavior change.

**Table 3:** Mobile Application Rating Scale scores.

Name	Engage	Function	Aesthetics	Information	Satisfaction	Behavior change	Overall
AskMD	5.0	5.0	5.0	4.6	4.3	4.5	4.9
WebMD	4.1	4.9	4.5	4.2	3.6	2.8	4.4
Symple	4.1	4.5	4.5	3.9	3.8	4.1	4.3
Heart Failure Health Storylines	4.3	4.2	3.3	4.5	3.9	4.6	4.1
Continuous Care Health App	4.1	4.6	3.7	3.7	4.1	3.8	4.0
Heart Keeper	4.0	4.0	4.0	3.5	3.0	4.2	3.9
mediSOS	3.8	4.3	3.7	3.8	3.5	3.5	3.9
Heart Log	4.5	4.5	3.0	3.2	2.0	2.8	3.8
Healthy Ally	3.5	4.0	4.0	3.5	2.5	3.0	3.8
MyHeartApp	3.9	3.5	4.3	3.3	3.1	4.1	3.7
Urgent Care 24/7	2.5	4.5	3.8	4.0	4.1	3.6	3.7
ASCVD Risk Estimator	3.0	3.8	3.2	4.3	2.3	3.2	3.6
FAQs in Heart Failure	2.5	4.3	3.7	3.8	2.1	2.8	3.6
Miniatlas Hypertension	2.1	4.5	3.7	3.8	1.9	2.5	3.5
iTriage	3.1	3.9	3.5	3.5	3.0	3.7	3.5
REKA	3.3	3.5	4.0	3.2	2.8	3.0	3.5
Heart Guide	2.5	3.5	4.7	3.0	2.0	4.0	3.4
SelfCare-MHR	2.8	4.3	3.3	3.3	2.3	3.0	3.4
WOW ME 2000mg	2.9	4.6	3.0	2.9	2.6	3.3	3.4
Cardiograph	2.9	3.8	3.5	2.8	1.8	3.8	3.2
BloodPressureDB	3.3	3.5	3.0	3.1	2.6	3.3	3.2
URI Life	3.5	3.0	3.0	3.0	2.3	3.0	3.1
Pulse Pro	2.1	3.4	3.2	3.1	2.1	2.5	2.9
Heart Disease & Symptoms	1.0	3.8	2.3	3.7	1.0	2.0	2.7
Health Manager	2.8	2.5	2.5	2.4	1.5	3.3	2.5
Heart Services	3.3	1.8	3.0	2.1	1.0	2.0	2.5
My Heart Rate Monitor & Pulse Rate	2.3	2.4	2.8	2.0	1.8	2.3	2.4
Heart Disease	1.2	4.1	1.6	2.2	1.3	1.5	2.2
My Cardiologist	1.3	1.3	3.0	2.3	1.0	1.3	2.0
iTreat-Medical Dictionary	2.0	1.5	2.3	1.2	1.0	1.0	1.7
My Health Tracker	1.5	2.0	1.0	2.3	1.0	1.3	1.7
Track your Heart Failure Zone	1.0	1.0	1.3	1.8	1.0	0.8	1.3
Healthy Heart Numbers	1.0	1.8	1.3	1.0	1.0	1.0	1.3
Healthy Heart	1.0	1.0	1.3	2.2	1.0	2.0	0.8

### Functionality

**Figure 2:**
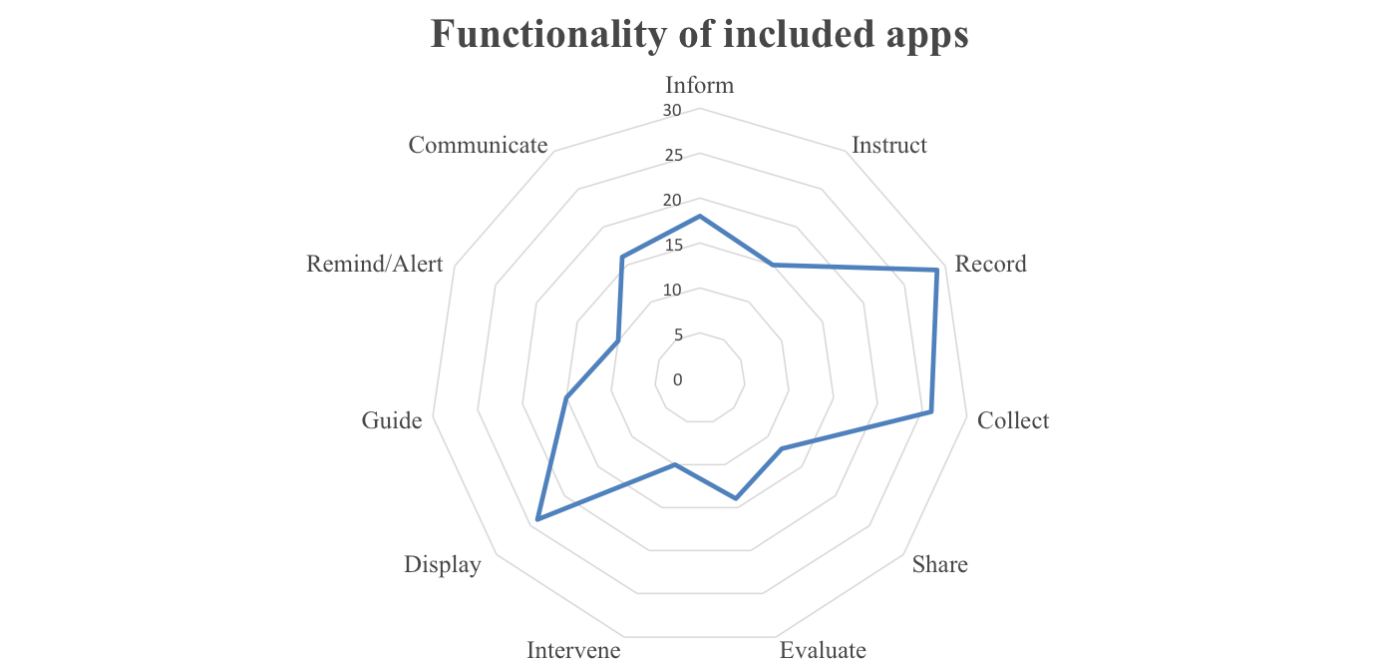
Functionality of included apps based on IMS Institute for Healthcare Informatics functionality scores.

Figure 2 illustrates the functionalities of the apps and highlights that nearly all had a record function (29/34, 85%). The median number of functionalities was 5 and the majority of apps (66%) had less than 7. Twenty-four apps had the option to display, 18 to inform, 16 to communicate, 15 to instruct, 15 to guide, and 10 to remind/alert. Three apps had a total of 11 functionalities (WebMD, Symple, and ContinuousCare Health App) followed by Heart Failure Health Storylines, which had 10 functionalities.

Of the 29 apps that had the function to record, 26 could collect data, 12 could share the data, and 10 had the function to intervene. Examples of data that were collected using these apps include medications, symptoms, daily moods, daily vital signs, and physical activity.

Heart Failure Health Storylines and WebMD have the ability to sync (collect) with a wide variety of fitness devices, apps, and even some scales. Many of the apps sync with the Apple Health app for iPhone users. Examples of apps that included the option to share data included Heart Failure Health Storylines and ContinuousCare Health App. In Heart Failure Health Storylines, the user has the option to share or communicate each feature with certain “circles of support” to which the user can add friends and family through email or keep the data private, including symptoms, vital signs, moods, and journal entries. Heart Failure Health Storylines does not have an option to share data using a message feature.

ContinuousCare Health App has a newsfeed feature that includes health-related articles and a customizable user profile. ContinuousCare Health App also includes the option for real-time consultation with a licensed health care provider, as well as a “Doctor Virtual Practice” that allows the user to invite his or her provider to virtually track data and communicate with the user in the app itself.

Symple offers a data exportation (share feature) that allows the user to back up data recorded in the app to a personal email or a spreadsheet app. In Symple users can also share an overview of current symptoms with their doctor. This document saves a PDF attachment and can then be sent over email. In WebMD the user can share health data and providers can respond and share education materials through this feature, which is password-protected.

### Heart Failure Society of America Guidelines

Table 4 includes the 8 HF-specific self-care behaviors evaluated. The most commonly addressed was daily monitoring of symptoms (21/34, 62%), followed by responding to symptoms (16/34, 47%), taking daily medications (13/34, 38%), following a low-salt diet (10/34, 29%), going to provider appointments (6/34, 18%), doing exercise (7/34, 21%), daily weighing (7/34, 21%), and checking extremities for swelling (8/34, 24%). The app that addressed all of these self-care behaviors was Heart Failure Health Storylines, which was developed in collaboration with the HFSA.

**Table 4:** Heart Failure Society of America–recommended nonpharmacologic management behaviors included in the apps.

Name	Weight	Check swelling	Physical activity	Diet	Medication	MD appointment	Monitor symptoms	Symptom response	Total score^a^
Heart Failure Health Storylines	✓	✓	✓	✓	✓	✓	✓	✓	8
WOW ME 2000mg	✓	✓	✓	✓	✓		✓	✓	7
WebMD	✓	✓	✓	✓	✓		✓	✓	7
ContinuousCare Health App	✓		✓	✓	✓		✓	✓	6
iTriage		✓		✓	✓		✓	✓	5
URI Life			✓	✓	✓	✓	✓		5
AskMD	✓	✓		✓	✓				4
Health Manager	✓				✓		✓	✓	4
Heart Keeper			✓		✓	✓	✓		4
MyHeartApp	✓			✓			✓	✓	4
Urgent Care 24/7		✓		✓			✓	✓	4
ASCVD Risk Estimator		✓	✓	✓					3
BloodPressureDB					✓		✓	✓	3
Symple		✓					✓	✓	3
Cardiograph							✓	✓	2
Heart Log						✓	✓		2
mediSOS					✓			✓	2
My Heart Rate Monitor & Pulse Rate							✓	✓	2
Pulse Pro							✓	✓	2
REKA							✓	✓	2
SelfCare-MHR							✓	✓	2
Healthy Ally							✓		1
Healthy Heart Numbers					✓				1
Heart Disease & Symptoms						✓			1
Heart Guide							✓		1
Miniatlas Hypertension					✓				1
My Cardiologist						✓			1
My Health Tracker							✓		1

^a^Apps that scored a zero did not include any symptom monitoring or self-care behaviors and were removed from the table.

### Overall App Quality

Factoring in the MARS, IMS Institute for Healthcare Informatics functionality, and HFSA guideline scores, the highest performing apps included Heart Failure Health Storylines, Symple, ContinuousCare Health App, WebMD, and AskMD.

A PubMed search of the apps in this review found that only 3 apps have been evaluated and published in peer-reviewed journals [30-32].

## Discussion

This study is the first to comprehensively review commercially available mobile apps for HF symptom monitoring and self-care and independently evaluate their quality using validated multiple rating scales, including the MARS expert rating scale, IMS Institute for Healthcare Informatics functionality scale, and HFSA guidelines for nonpharmacologic management. The most common functionality among the 34 apps reviewed was being able to record information, typically syncing data from other sources. Few apps provided any guidance in response to reported input, reminders or alerts about medications or symptom tracking, or the ability to communicate with providers.

**Figure 3:**
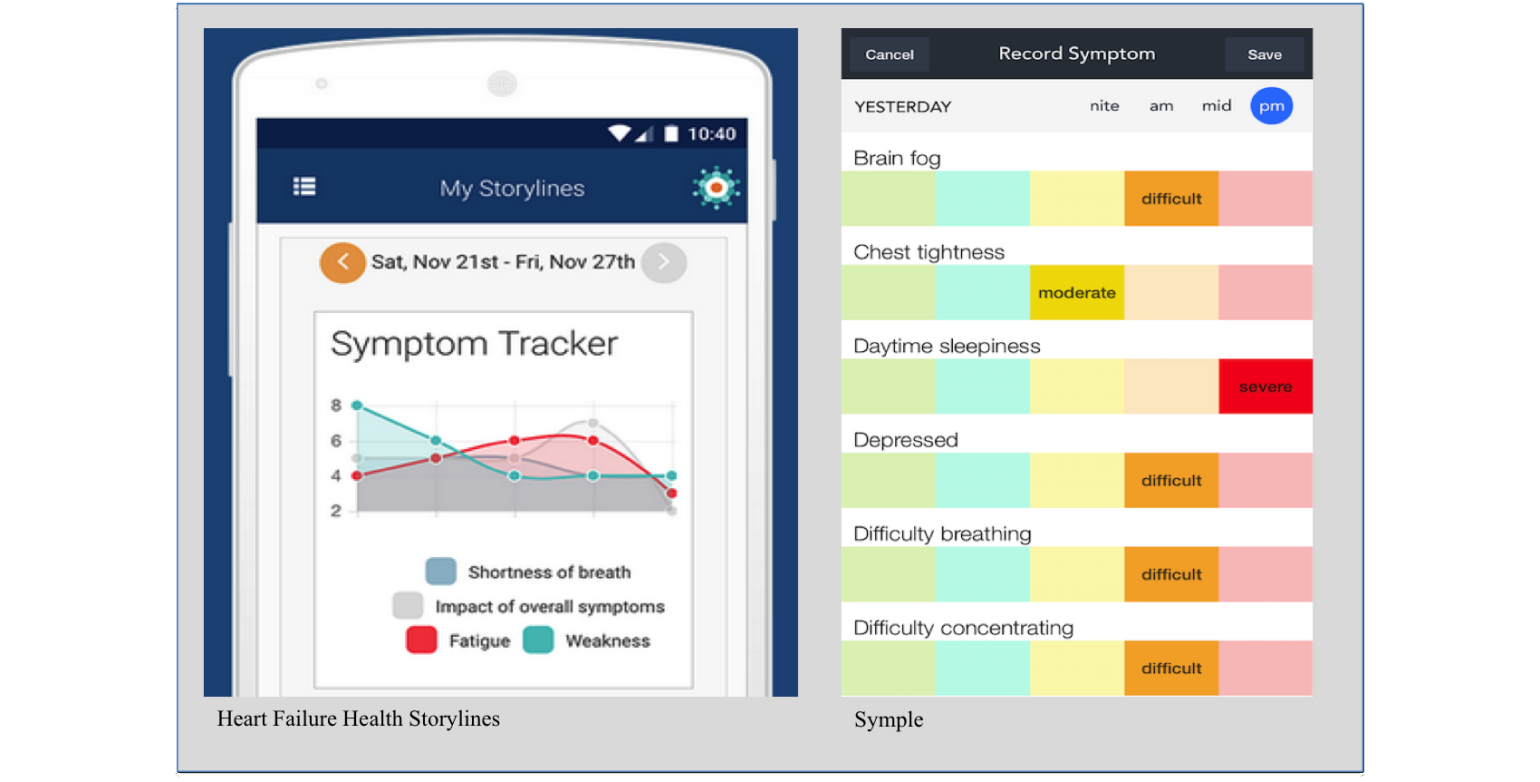
Symptom tracking features. Heart Failure Health Storylines (left image) enables tracking symptoms over time and Symple (right image) enables reporting symptoms for a single day and visualization of individual graphs by symptom.

The 2 apps that provided the most options for symptom tracking included Heart Failure Health Storylines and Symple (Figure 3). Heart Failure Health Storylines includes the feature of being able to track multiple symptoms simultaneously to allow the user to detect potential correlations between symptoms and time periods. Users can record symptom severity, moods, vital signs, and medications and the data are displayed using a color-coding scheme and weekly calendar format. The vital sign data are also displayed in a line graph to show daily fluctuations.

In Symple, users enter symptoms by calendar date and select the time of day and severity of each symptom (none, mild, moderate, difficult, severe) from a color-coded graphic (Figure 3). Symptoms are displayed using blocks with the same color-coding scheme in a weekly calendar format. Users can choose to view symptoms from one time of day (eg, every morning this week) or the entire day. One drawback of this feature is that it only allows viewing of one symptom at a time; multiple symptoms cannot be plotted on the same calendar.

Despite the pressing need that patients with HF have for better symptom and self-monitoring tools, most mHealth apps are designed to support healthy living rather than chronic disease management. Many apps focused on helping patients find a diagnosis for their symptoms (ie, AskMD and WebMD). Some of the apps supported self-care maintenance in terms of recording daily health behaviors or including reminders about taking medication, but were very limited with self-care management behaviors including more advanced symptom monitoring, tracking, and evaluation of whether specific behaviors improved health outcomes.

A total of 3 peer-reviewed articles evaluated 3 of the Web-based apps. The first article was a brief review on the development and future directions of the ASCVD Risk Estimator app [30]. The second article was an evaluation of the Heartkeeper app using Google app usability standards (completed by the authors) and a quality of experience survey completed by 24 users who were recruited through the app itself [31]. This review found the app to be generally compliant with Google’s standards; it had mixed feedback on the quality of experience. The third article was a 4-month trial of the iTreat app in the hospital setting among 39 junior doctors in the United Kingdom [32]. Although participants reported some positive outcomes from using the app, the study highlighted many barriers to the use of mobile phones in the hospital setting [32].

Many apps are being used with minimal knowledge of their functionality and ability to integrate data into health care systems [27], let alone efficacy for improving patient or clinical outcomes. The lack of efficacy testing in clinical trials is one of the biggest barriers to adoption of mHealth apps. Health care providers are reticent to prescribe apps without evidence of their benefit, guidelines regarding use in clinical practice, and confidence in the privacy and security of personal health information that is both stored and transmitted [27]. These barriers are the major reasons why apps need to undergo rigorous clinical trial testing before they can be fully integrated into clinical care.

One good example of an mHealth app with demonstrated effectiveness for managing diabetes is BlueStar from WellDoc Diabetes Management. This app has been evaluated in a clinical trial and has demonstrated effectiveness for supporting diabetes management [33]; however, it is only accessible for patients with diabetes who have a prescription from their health care provider and it is not otherwise available to the public.

These findings suggest that apps have not yet been readily adopted into routine clinical management and need further development to support comprehensive symptom management for patients with HF. The limited number of apps and functionalities of specific apps targeting HF behaviors suggests that the apps are in an early stage of development and that patients and providers who would be using them are at an early stage of adoption. This is also true for some older adults who have lagged behind in the adoption of smartphone technology as well [11]. One of the ongoing priorities for the adoption of mHealth apps into clinical practice will be the rigorous assessment of app quality as demonstrated in this study and effectiveness in rigorous comparative effectiveness studies. Improving the ability of apps to engage is also a targeted area for future improvement.

### Strengths and Limitations

Strengths of this review include applying a rigorous multistep methodology to the evaluation of the apps and using the MARS rating scale. The star rating system can be misleading given the low numbers of ratings that some of these apps have. Systematic consolidation and rigorous evaluation beyond the star rating system and user comments are needed for patients to be able to evaluate which apps may be best for their symptom monitoring and self-care. The use of the MARS was a strength because it was rigorously developed [26] and has been used to evaluate apps related to mindfulness [34] and weight management [35]. One of the limitations of this review is that apps that were not publically available were not included, such as those that required a prescription or enrollment in a specific health care network or insurance plan.

### Future Research

According to a report by the IMS Institute for Healthcare Informatics, one of the most important areas for future research will be the generation of evidence of value from the use of apps that will demonstrate the magnitude of behavior change and improved health outcomes [27]. Evaluation of existing apps should use rigorously tested tools, such as the MARS or IMS functionality score. In addition, future studies should test the effectiveness of apps with higher functionality and usability scores. Further mapping of HF-specific apps to evidence-based clinical guidelines is needed. Focusing on improving apps that are already commercially available is a viable option.

In addition, there is also the need for enhanced interoperability between electronic health records and apps so that real-time data can inform clinician decision making and clinical management. Enhanced data integration should take place within the context of robust organizational governance frameworks that take into consideration the evaluation of clinical outcomes [36].

### Conclusions

In general, mHealth apps offer a potentially cost-effective solution with 24/7 access to symptom monitoring at the point of need, promotion of patient engagement in their care, and can foster interactive care and communication with providers. Increasing the options for mHealth apps to support successful care management is critical. Patient collaboration with health care providers and decision making is a core component of patient engagement [37,38], improving quality of life and decreasing hospital use [39]. Our review highlights the need for further refinement and mapping to guidelines and room for overall quality improvement in HF symptom monitoring and self-care related apps. To ensure engagement, ease of use, and aesthetics, patients also need to be involved in the development of the mHealth apps.

## Abbreviations

CA-ICC: consistency-of-agreement intraclass correlation
HF: heart failure
HFSA: Heart Failure Society of America
ICC: interclass correlation coefficient
MARS: Mobile Application Rating Scale
mHealth: mobile health
